# Supplementary material for: Towards unified AI-driven fracture mechanics: the extended deep energy method (XDEM)
Source: Nat Commun. 2026 Aug 15;17:8492. doi: 10.1038/s41467-026-76748-1 (PMC13484471; doi:10.1038/s41467-026-76748-1)
Supplement: Supplementary file 3 [file 41467_2026_76748_MOESM3_ESM.zip › pic/Results/Crack_propagation/Bittencourt/Figure_4.pdf]

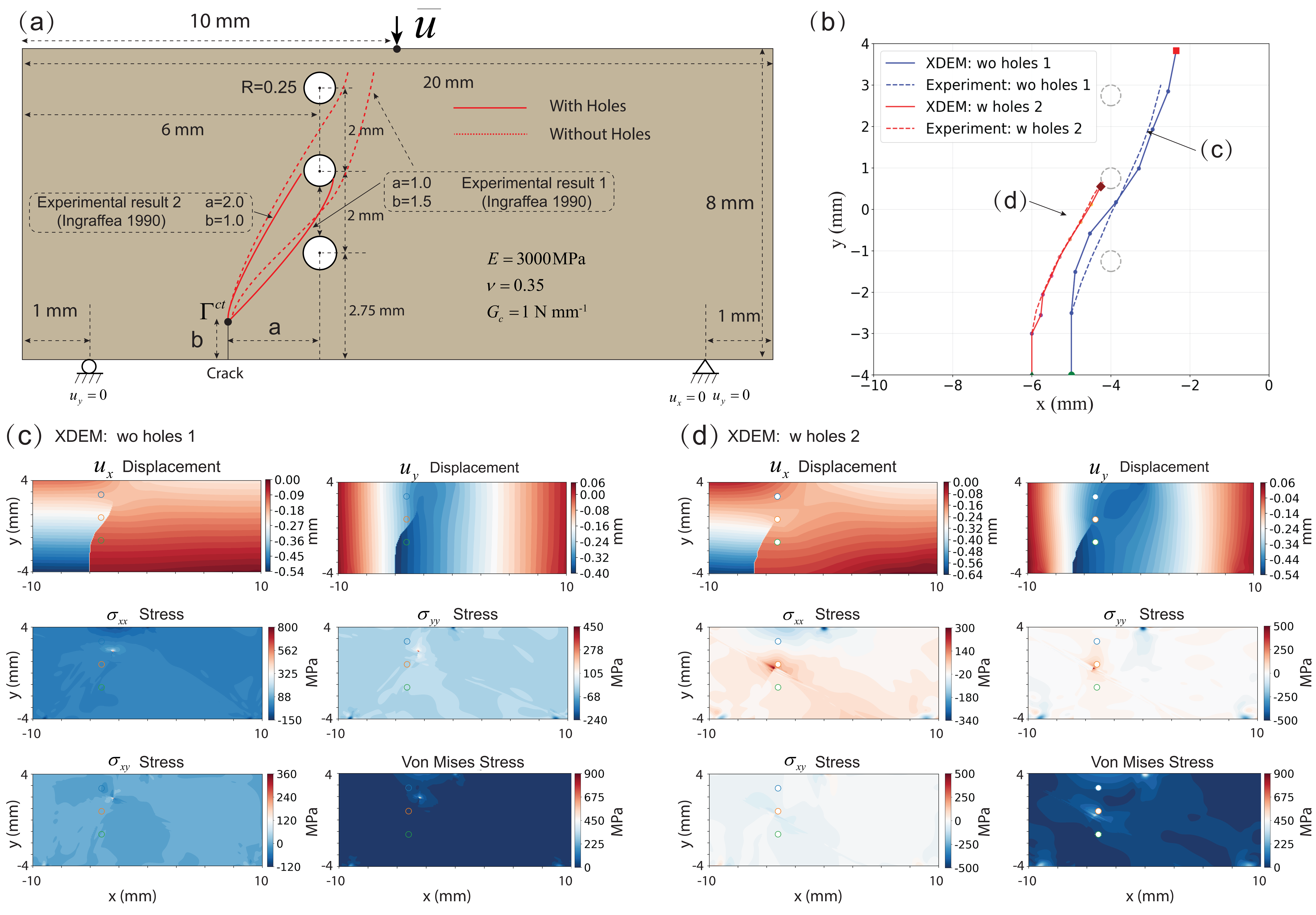

(a)
10 mm
$\bar{u}$
20 mm
R=0.25
6 mm
2 mm
2 mm
2.75 mm
With Holes
Without Holes
Experimental result 2 (Ingraffea 1990) a=2.0 b=1.0
a=1.0 b=1.5 Experimental result 1 (Ingraffea 1990)
8 mm
$E = 3000$ MPa
$\nu = 0.35$
$G_c = 1$ N mm^{-1}
1 mm
1 mm
Γ^{ct}
a
b
Crack
$u_y = 0$
$u_x = 0$ $u_y = 0$
(b)
XDEM: wo holes 1
Experiment: wo holes 1
XDEM: w holes 2
Experiment: w holes 2
(c)
(d)
x (mm)
y (mm)
(c) XDEM: wo holes 1
(d) XDEM: w holes 2
u_x Displacement
u_y Displacement
σ_{xx} Stress
σ_{yy} Stress
σ_{xy} Stress
Von Mises Stress
mm
MPa
